# A Prospective Evaluation of the Association of Ureteral Wall Thickness With Intraoperative Stone Impaction in Ureteroscopy

**DOI:** 10.7759/cureus.35972

**Published:** 2023-03-10

**Authors:** Yasir Rasheed, Syed M Nazim, Kaleem K Mirani, Maheen Zakaria, Muhammad Bin Nasir

**Affiliations:** 1 Surgical Oncology, Shaukat Khanum Hospital, Lahore, PAK; 2 Urology, Aga Khan Hospital, Karachi, PAK; 3 Surgery, Aga Khan University Hospital, Karachi, PAK; 4 Urology, Aga Khan University Hospital, Karachi, PAK; 5 School of Medicine, Aga Khan University, Karachi, PAK

**Keywords:** non-contrast computed tomograpgy, stone clearance rate, ureteral wall thickness, stone impaction, urs

## Abstract

Background and objective

In this study, we aimed to analyze the association of ureteral wall thickness (UWT) measured on non-contrast CT (NCCT) with stone impaction as found in ureteroscopy (URS).

Materials and methods

We analyzed 43 patients who underwent URS and pneumatic/laser lithotripsy for ureteric stones from May to November 2022. The UWT was measured by an experienced radiologist on NCCT. Clinical predictors of the impacted stone were calculated by univariate and multivariate regression analysis. The receiver operating characteristic (ROC) curve was calculated for the UWT cutoff to apply it for impaction with different parameters. We also evaluated the association of intra- and postoperative parameters of the two groups with UWT.

Results

Out of the 43 patients with stones, 26 (60.46%) patients had impacted stones. Univariate analysis was used to analyze the site (left-sided stone impacted more commonly), stone size, stone density [Hounsfield unit (HU)], hydronephrosis, UWT, and duration between initial presentation and surgery, and multivariate analysis was utilized to assess stone density, as well as UWT's association with impacted stones. The ROC curve showed a cutoff of 3.5 mm for UWT with an accuracy of 0.83. High UWT (≥3.5 mm) was associated with a significantly lower stone-free rate, more complications, and mean operative time as compared to low UWT (<3.5 mm) (p<0.05).

Conclusion

Based on our findings, high UWT is associated with high rates of impacted stones and a lower stone-free rate when compared to low UWT.

## Introduction

Urologists commonly encounter ureteral stones in their daily practice. Many modalities of treatment, such as ureteroscopy (URS), shock wave lithotripsy (SWL), and percutaneous nephrolithotomy (PCNL), are used for their management depending on various stone and patient parameters [1]. During URS, it is not uncommon to encounter impacted stones in the ureter, which poses various risks and challenges. Stone impaction leads to inflammation in ureteral mucosa with resulting hypertrophy, edema, polyp formation, and fibrosis at the site of impaction with the adhesion of stone to the ureteral wall [2]. This leads to intraoperative difficulty in stone removal as well as lower chances of spontaneous passage of residual fragments. The management, perioperative parameters, and postoperative outcomes vary significantly with respect to impacted stones when compared to non-impacted stones. In impacted stones, the operative time, failure rate, and chances of complications including sepsis, are significantly higher [3]. Identification of factors that can predict stone impaction preoperatively could help not only in better planning of the surgery but also in counseling the patients about the various potential clinical outcomes, such as chances of the success of the procedure, risk of complications, and the need for ancillary procedures. In a recent study, Yoshida et al. retrospectively analyzed 130 URS procedures and found ureteral wall thickness (UWT) measured on non-contrast CT (NCCT) to be a good predictor of impacted stones in URS. They identified a cutoff of 3.49 mm for UWT with regard to the predictability of the impaction of ureteral stones during URS [2].

Sarica et al., in a prospective study, evaluated the management of impacted proximal ureteric stones with semi-rigid URS and found that the chances of finding residual stones, the need for the placement of JJ stents, and operative time were higher in patients with greater UWT [4] Other studies have also linked UWT with predicting the spontaneous stone passage and the success of internal ureteral stent placement and extracorporeal shock wave lithotripsy (ESWL) [5].

This study aimed to analyze the association of UWT measured on NCCT scan with stone impaction as found in URS. We also calculated differences in intraoperative and postoperative outcomes with respect to UWT.

## Materials and methods

This study was conducted prospectively for a period of six months at our tertiary care hospital from May 2022 to November 2022. The study protocol was approved by the Institutional Ethical Review Committee (ERC) (ref #2022-7364-21529). A minimum sample size of 32 patients (with ureteral stone) was calculated using the software OpenEpi considering the mean difference of 1.69 mm in UWT between the population having impacted and those with non-impacted stones and to achieve 80% power at an alpha 0.05 [4].

All patients aged >18 years who underwent elective day care procedures with semi-rigid URS for a single, radiopaque, unilateral ureteral stone confirmed on NCCT scan were included. Patients with congenital renal anomalies, solitary kidney, renal insufficiency, untreated coagulopathy, previously diagnosed ureteral stricture, or those who had undergone prior interventions (e.g., JJ stenting/percutaneous nephrostomy) for the same stone were excluded.

Patients underwent standard procedures such as detailed history taking, examination, and standard investigations. Our study did not change any standard process already in place for the evaluation of ureteral stones. An NCCT scan was performed using a 640-slice scanner (Aquilion, Toshiba Medical Systems TM, Shimoishigami, Otawara-Shi, Japan), employing 3-mm axial and reformatted 3-mm coronal sections. The images were evaluated on a picture-archiving computer application (View Pro-X version 4.0.6.2; Rogan-Delft, Veenendaal, Holland). All NCCT scans in our setup had a scout film that was used to see if the stone was radiopaque or not. This helped with subsequent follow-up with an X-ray to look for stone clearance. Stone-related parameters such as stone size, location in the ureter, and Hounsfield units (HU) were recorded. Stones were classified to be either in the upper, mid, or lower ureter. Calculi above the sacroiliac joint were labeled as upper ureteral stones, those anterior to the sacroiliac joint were considered mid-ureteral, and those below the sacroiliac joint were considered lower ureteral. We also noted the degree of hydronephrosis and severity graded as per the Society for Fetal Urology grading system, if present. HU was calculated by taking a mean of the HU recorded at three different locations of the stone. Stone size was also noted in two dimensions: one along its maximum visualized diameter and the other one perpendicular to it. This approach gave us an estimate of the stone area [6], based on the following formula:

Stone area = maximum diameter x perpendicular diameter.

**Figure 1:**
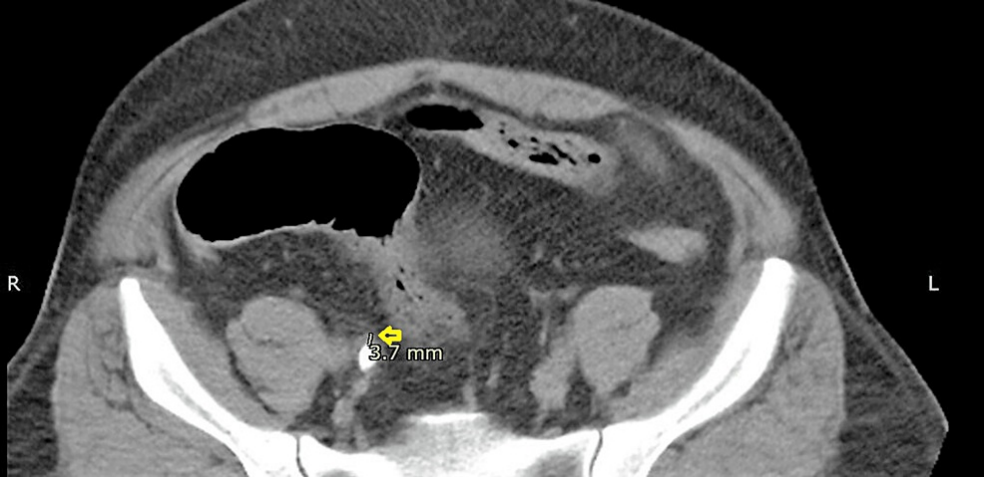
NCCT scan - axial image The image shows a right ureteric stone with a UWT of 3.7 mm. The arrow indicates UWT NCCT: non-contrast computed tomography: UWT: ureteral wall thickness

UWT was measured as the point of highest soft-tissue thickness, i.e., ureteral wall + periureteral edema surrounding the stone (from top to bottom of stone) on NCCT axial image on standard soft tissue window setting, as shown in Figure 1.

Demographic data were recorded. Patient-related factors such as age, gender, body mass index, comorbidities, and duration from the start of symptoms related to the stone to the day of surgery were documented.

All patients underwent URS under general anesthesia as a primary treatment procedure in the lithotomy position using a 6.4/8.0 Fr semi-rigid Karl Storz TM ureteroscope, with the aid of fluoroscopy. Stone fragmentation was done using a pneumatic lithoclast (Swiss lithoclast TM Master, EMS, Nyon, Switzerland) or Ho: YAG laser with 360-micron fiber. Before starting URS, the ureteric orifice was cannulated in all patients using a standard (0.038") guidewire (Boston Scientific, Marlborough, MA) under fluoroscopic guidance and an attempt was made to pass it beyond the stone. Impacted stone was defined and recorded as a stone through which this guidewire did not pass in the initial attempt [2,7]. Intraoperatively, endoscopic findings such as stone impaction, any kinks in the ureter, mucosal edema, polyp, and ureteric stricture were noted. Any iatrogenic ureteric mucosal injury or perforation/contrast extravasation was also recorded. At the end of each procedure, stone-free status was noted. A patient was classified as stone-free on the basis of intraoperative endoscopic findings; fluoroscopy at the end of the procedure; and kidney, ureter, and bladder (KUB) X-ray performed two weeks postoperatively. Postoperative complications were recorded as per the modified Clavien grading system, which ranged from grade 1 to grade 5 [8].

## Results

A total of 113 semi-rigid ureteroscopies were performed during the study period in our setup. Out of these, 43 patients were included in the final analysis. The rest were excluded due to the following reasons: 23 had an NCCT from outside our hospital with collimation more than 3 mm, 15 patients did not get a follow-up X-ray, seven had prior JJ stent in place at the time of presentation, and 12 had more than one stone in the ureter while 13 had radiolucent stones. According to the definition provided above, among the 43 patients included in the study, 26 (60.46%) patients had an impacted stone and 17 (39.54%) did not. Table 1 shows the characteristics of the patients and the stones. Patients with left-sided stones were more at risk of impaction in our study population (p=0.006). Stone size was significantly different in impacted (7.99 ± 3.32 mm) vs. non-impacted (5.57 ± 2.36 mm) groups (p=0.013). Ureteral wall thickness was also significantly different between the two groups: 4.6 ± 1.41 mm in the impacted vs. 2.55 ± 1.26 mm in the non-impacted group (p<0.001). The other factors that were significant between impacted vs. non-impacted ureteral stones were stone HU (p=0.006), duration between initial clinical symptoms and surgery (average: 28 days; 42 days in patients with impacted stones vs. 14 days in patients without impacted stones; p=0.012), degree of hydronephrosis (in the group with impacted stones, none had mild hydronephrosis, while 30% had moderate to severe hydronephrosis; in patients without impacted stones, eight patients had no hydronephrosis, nine had mild hydronephrosis, while none of the cases had moderate or severe hydronephrosis; p<0.001).

**Table 1:** Demographics and preoperative characteristics of the cohort IQR: interquartile range; SD: standard deviation

Variable		Overall (n=43)	Patients with impacted stones (n=26)	Patients without impacted stones (n=17)	P-value
Age, years		37 (IQR=21)	45.0 (SD=12.9)	37.1 (SD=12.1)	0.050
Gender, n (%)	Male	32 (74.4%)	20 (62.5%)	12 (37.5%)	0.728
	Female	11 (25.6%)	6 (54.5%)	5 (45.5%)	
BMI, kg/m^2^		27.54 (SD=3.82)	26.9 (SD=3.3)	28.4 (SD=4.4)	0.217
Sites, n (%)	Left	26 (60.5%)	20 (76.9%)	6 (23.1%)	0.006
	Right	17 (39.5%)	6 (35.3%)	11 (64.7%)	
Stone location, n (%)	Proximal ureter	16 (37.2%)	8 (50%)	8 (50%)	0.473
	Middle ureter	8 (18.6%)	6 (75%)	2 (25%)	
	Distal ureter	19 (44.2%)	12 (63.2%)	7 (36.8%)	
Stone size, mm		7.03 (SD=3.18)	7.99 (SD=3.32)	5.57 (SD=2.36)	0.013
Stone area, mm^2^		32.68 (IQR=30.80)	35.88 (IQR=40.16)	20.00 (IQR=24.79)	0.09
Hounsfield units		750 (SD=250)	831.9 (SD=244.4)	624.4 (SD=208.7)	0.006
Ureteral wall thickness, mm		3.79 (SD=1.68)	4.60 (SD=1.41)	2.55 (SD=1.26)	<0.001
The duration between initial clinical signs and surgery, days		28 (IQR=31)	42.0 (IQR=39.0)	14.0 (IQR=24.5)	0.012
Hydronephrosis, n (%)	None	8 (18.6%)	0 (0%)	8 (30.8%)	<0.001
	Mild	27 (62.8%)	18 (66.7%)	9 (33.3%)	
	Moderate	5 (11.6%)	5 (19.2%)	0 (0%)	
	Severe	3 (7%)	3 (11.5%)	0 (0%)	

We evaluated factors related to impacted stones using multiple logistic regression analysis by employing significant components from the univariate analysis (Table 2).

**Table 2:** Multivariate logistic regression analysis of the cohort OR: odds ratio; CI: confidence interval

Variable	OR (95% CI)	P-value
Age	0.95 (0.901-1.001)	0.056
Stone area	0.97 (0.94-1.004)	0.09
Stone size	0.79 (0.553-1.131)	0.198
Stone density	0.996 (0.992-0.999)	0.015
Ureteral wall thickness (UWT)	0.299 (0.144-0.619)	0.001

**Figure 2:**
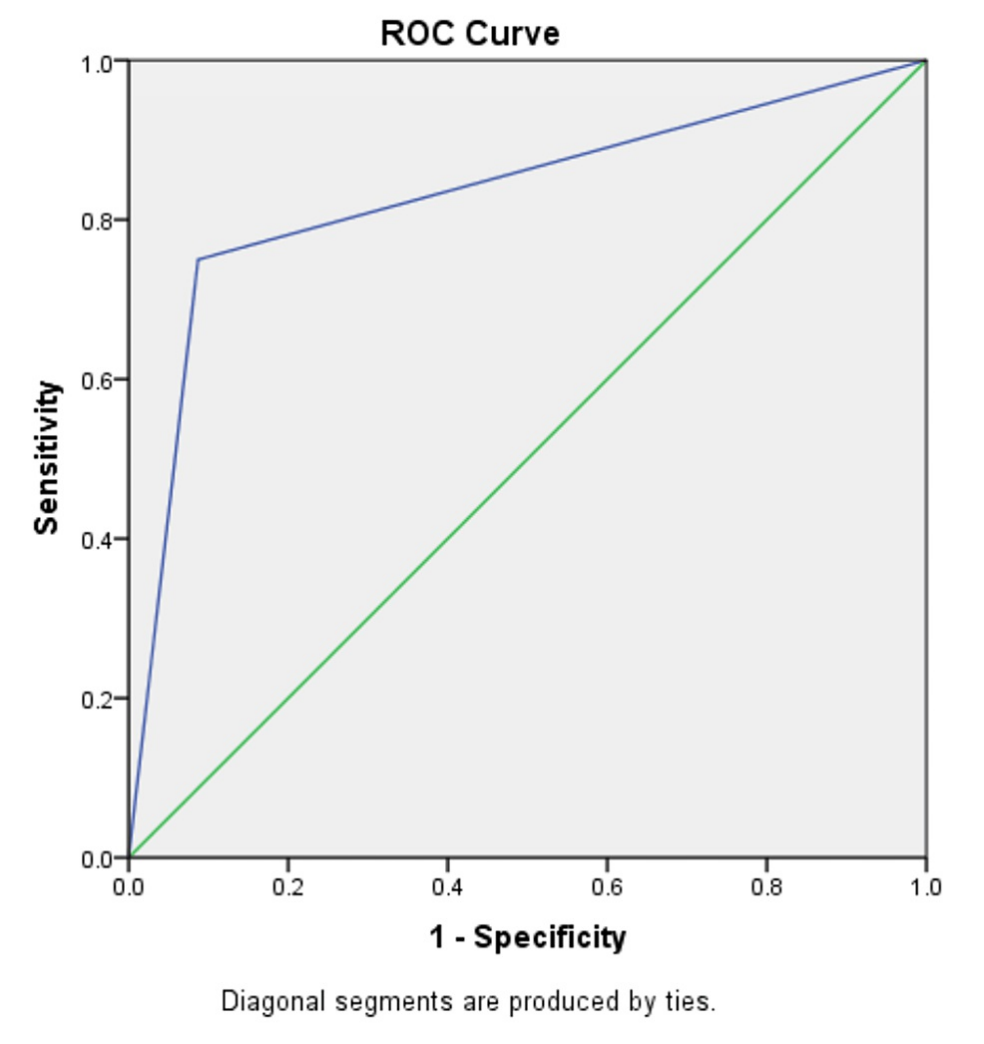
ROC curve for UWT ROC: receiver operating characteristic; UWT: ureteral wall thickness

We found UWT (OR: 0.299, p=0.001) and stone density (OR: 0.996, p=0.015) to be reliable indicators of the presence of impacted stones. Additional variables, such as age, stone size, stone area, and stone position were also analyzed, but no significant associations were found. A UWT of 3.50 mm was determined to be the optimal cutoff value for predicting impacted stone by receiver operating characteristic (ROC) curve analysis, with an area under the curve (AUC) of 0.83 (95% CI: 0.69-0.96). This cutoff exhibited an 87% specificity rate and a 75% sensitivity rate for predicting impacted stones (Figure 2).

As shown in Table 3, we classified the cases into two groups based on the cutoff value of 3.50 mm: those with high UWT (≥3.50 mm) and those with low UWT (<3.50 mm). We then compared endoscopic findings and surgical results between the two groups. There was a substantial difference in stone clearance across all measures, with 90% clearance in patients with UWT <3.50 mm and 69% clearance in patients with UWT ≥3.50 mm (p=0.001). A significant difference in operative time difference was also found between the two groups, with a mean operative time of 51 minutes in patients with high UWT (≥3.50 mm) and 39 minutes in patients with low UWT (<3.50 mm) (p=0.03). Patients with UWT ≥3.50 mm had a higher incidence of complications and requirement for stenting, but this disparity was statistically negligible. Out of six patients in whom different complications were seen in the high UWT group, three patients underwent ancillary procedures, two patients underwent re-do URS at a later stage, and one patient underwent ESWL. Also, four patients in the high UWT group were admitted from surgical daycare after the procedure while none of the patients in the contralateral group were admitted.

**Table 3:** Association of UWT with endoscopic findings and intra- and postoperative outcomes SD: standard deviation; UWT: ureteral wall thickness

Variable	<3.5 mm	≥3.5 mm	P-value
Endoscopic findings (ureteral edema, polyp, stricture), n (%)	3 (15%)	8 (34.78%)	0.003
Complications (ureteral injury/perforation/contrast extravasation), n (%)	2 (10%)	6 (26.08%)	0.006
Stenting, n (%)	13 (65%)	16 (69.56%)	0.07
Stone clearance, n (%)	18 (90%)	16 (69.56%)	0.001
Mean operative time in minutes (SD)	39.20 (14.04)	51.52 (21.25)	0.03

## Discussion

Acute loin pain is frequently brought on by urolithiasis, which is becoming more common and has significantly burdened healthcare systems globally in recent years [9,10]. Based on previous studies, 75-90% of ureteric stone passages occur spontaneously [11]. The first treatment option is watchful waiting, with or without concurrent medical expulsive therapy (MET), if the stone is anticipated to pass naturally within a fair amount of time and the symptoms remain tolerable [12]. ESWL, laser lithotripsy, or PCNL are used to treat stones that are not likely to pass spontaneously [13]. Urologists commonly encounter impacted stones during URS [3]. Stone impaction results in an inflammatory response in the ureter's mucosa, which leads to edema, mucosal hypertrophy, and scarring [14]. It is a time-sensitive process due to the embedding of calculi in the ureteral wall and can affect not only their spontaneous passage but also the outcomes of endo-urological procedures.

There are several definitions of stone impaction. A stone is considered "impacted" if it stays at the same location for an extended amount of time [3]; however, the exact day of impaction and thus the duration is difficult to determine. Another indicator of stone impaction is failure to visualize the ureter distal to stone on intravenous pyelogram (IVP) or contrast-enhanced CT [15]. However, these modalities are not routinely used in the setting of urolithiasis and have limitations of use in patients with renal insufficiency or contrast allergy. Another definition pertains to the inability of the guidewire to pass past the stone on the initial attempt [16]. We employed the latter definition of stone impaction in our study.

Numerous variables that influence stone impaction have been extensively researched in the literature. These variables, which were observed to considerably correlate with stone impaction, include sex, ASA score >1, positive urine culture, past same-side surgery, stone diameter, stone position, hydronephrosis, and UWT [13,16]. The average UWT is around 1 mm [17], and any value beyond it with stone increases the possibility of impacted stone; UWT increases with respect to the severity of ureteral obstruction depending upon the time elapsed and degree of inflammatory reaction. This parameter can be assessed on NCCT, which is a noninvasive way to assess the degree of stone impaction and thereby the obstruction caused by the stone. 

There is considerable data in the literature addressing stone impaction and its association with both intraoperative and postoperative difficulties. Different studies have set various cutoff values, which are pertinent in terms of counseling patients about significant impaction. This could translate into the risk of complications during URS, failure of stone retrieval, and the need for additional treatment. Yoshida et al. identified UWT measured on NCCT as a predictor for spontaneous stone passage of ureteral stones <10 mm. Their analysis revealed 2.71 mm as the optimal cutoff value for UWT with a predictive accuracy of 0.83 [18]. Samir et al. also correlated UWT with the spontaneous stone passage in uncomplicated distal ureteral stones in 212 patients. Their analysis showed a UWT cutoff of 3.75 mm as a potential predictor for spontaneous passage [19]. Similarly, another study has identified UWT as a reliable factor for not only stone impaction but also surgical outcomes in patients undergoing URS. The cutoff value was 3.49 mm with a predictive accuracy of 0.87 [2]. Sarica et al. identified UWT as a critical predictor for the success rate of impacted ureteral stones treated by SWL. Their study identified a cutoff of 3.55 mm on NCCT with an accuracy of 0.924 [20]. Kirli et al. retrospectively analyzed the role of UWT in predicting stone-free rates and complications in 147 children undergoing semi-rigid URS for ureteral calculi and found an optimal cutoff value of 4.5 mm to predict surgical outcomes [21].

We investigated UWT size in our population and discovered it to be an independent variable for stone impaction. Although the mean UWT in our population was 3.79 mm, it was considerably high (4.6 mm) in patients with impacted stones compared to 2.55 mm in patients with non-impacted ones. We identified a cutoff value of 3.5 mm for predicting impacted stones with an AUC of 0.83 on ROC analysis. This could preoperatively help prepare urologists with regard to anticipating stone impaction, intraoperative changes as a result of impaction, bleeding, and greater morbidity and mortality risk. In addition, it also assists urologists in counseling patients about the potential consequences of failure, as well as the need for stenting and referral to stone experts [22].

Regarding URS in patients with impacted stones, one can plan beforehand and keep hydrophilic-coated guidewires ready in the operating room since PTFE-coated guidewires are more likely to cause ureteric injury [23] and, in cases of large proximal ureteric impacted stones, one can consider an antegrade approach to reduce the risk of ureteral perforation and ureteral stricture, and thereby achieve better clearance [24].

Sarica et al. in another study determined the predictive value of UWT regarding the success of internal ureteral stent placement in patients with unilateral solitary obstructing stones and found a cutoff UWT value of 3.35 mm [25]. Recently, Abdrabuh et al. published a study where they evaluated the role of UWT in predicting ureteral stone impaction stratified according to the stone size and proposed a cutoff value of 3.8 mm for stones <10 mm and 4.1 mm for stones >10 mm. They proposed that this size stratification of UWT is a more practical clinical approach for predicting stone impaction [26]. Özbir et al. proposed an “impacted stone formula (ISF)” based on variables identified in preoperative NCCT findings. This formula includes not only UWT but also HU of the center of the ureter distal and proximal to the stone and the grade of hydronephrosis. The authors claimed that ISF is the most precise preoperative predictor of impacted ureteral stones by far [16].

Many studies on UWT are retrospective in nature; however, our study was prospective. We set a cutoff of UWT with an AUC of 83%, which is lacking in many previous studies on the subject. UWT was identified as an independent factor in both univariate and multivariate analyses. The limitations of the study include its non-randomized nature and the fact that this was a relatively underpowered study due to the smaller sample size. Moreover, we did not take into account intra- and interobserver differences, as well as long-term complications such as ureteral strictures.

## Conclusions

Based on our findings, UWT is a reliable predictor of stone impaction in the ureter and subsequently increases the risk of intraoperative and postoperative complications and that related to stone clearance. By applying the optimal cutoff value of UWT, urologists can counsel patients more efficiently about potential complications, the need for admissions as inpatients postoperatively, and requirements for ancillary procedures, thereby better preparing patients and themselves for URS.
